# Zoonotic vascular endograft infections are rare but serious complications

**DOI:** 10.1016/j.jvscit.2025.101938

**Published:** 2025-07-28

**Authors:** Andras Bikk, Almira Opardija, Lauren Johnson, Amin Joseph, Ekam Sekhon, Viraj Pandit

**Affiliations:** a Department of Surgery, Veterans Affairs-Central California Health Care System, Fresno, CA; b Department of Infectious Disease, University of California – Fresno, Fresno, CA; c Department of Surgery, University of California San Francisco, San Francisco, CA

**Keywords:** Atypical, Bartonella, Cat-scratch disease, Coxiella, Endograft, Infection, Q-fever, Vascular, Zoonotic

## Abstract

Zoonotic infections—bacterial, viral, fungal, or parasitic—can spread from domestic or wild animals to humans, either directly or via intermediate vectors. In vascular and endovascular surgery, infections are rare and usually caused by common bacteria with familiar presentations. In contrast, zoonotically transmitted, atypically behaving organisms pose diagnostic and therapeutic challenges due to their elusive nature and resistance to conventional detection methods. We present two rare cases of aortic endograft infections caused by *Coxiella burnetii* and *Bartonella henselae*. This case series highlights the importance of early suspicion, targeted serologic and molecular diagnostics, and the role of 18F-fluorodeoxyglucose positron emission tomography/computed tomography in diagnosis.

Zoonotic diseases—transmitted from animals to humans—include a wide spectrum of pathogens. Although *Coxiella burnetii* and *Bartonella henselae* are recognized causes of culture-negative endocarditis,^1^, ^2^, ^3^ their role in vascular infections, particularly endograft involvement, is far less documented.

Vascular infections, especially those involving endografts, are uncommon.^4^^,^^5^ When present, they are typically due to familiar pathogens with identifiable signs. Atypical organisms like *Coxiella* and *Bartonella*, however, are less readily suspected and detected, leading to potential delays in diagnosis and treatment.

*C. burnetii* is a Gram-negative, obligate intracellular bacterium, transmitted primarily via inhalation of aerosolized particles from infected animals, particularly ruminants.^6^^,^^7^ Chronic Q fever can manifest months after exposure, with vascular infections seen in 7% to 10% of chronic cases—often involving the abdominal aorta or grafts.^8^^,^^9^

*B. henselae*, a Gram-negative intracellular pathogen best known for “cat scratch disease,” is carried by domestic cats and transmitted via scratches or flea vectors.^10^^,^^11^ Although commonly self-limiting, rare complications include endocarditis and infection of the arteries, including the aorta.^12^, ^13^, ^14^

This manuscript presents two cases of zoonotic endograft infections managed at a Veterans Affairs hospital. The goal is to raise awareness of these pathogens, highlight diagnostic challenges, and propose management strategies for vascular surgeons. Patients signed a consent to publish images without personal identification.

## Case reports

### Case 1 – *Coxiella burnetii* infection

**Fig 1:**
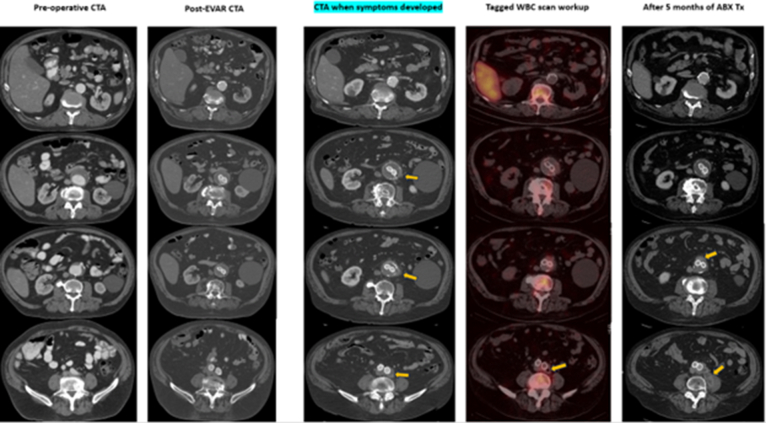
Captured timeline of the delayed development of *Coxiella burnetii* endograft infection after endovascular aortic repair (EVAR). *Arrows* marking retroperitoneal thickening, fat stranding, and enlarged lymph nodes next to the aorta at the time of initial symptoms. Tagged white blood cell (*WBC*) scan was negative. Antibiotic therapy (*ABX Tx*) was ineffective, and the infection continued to spread locally. *CTA*, Computed tomography angiography.

A 77-year-old man with a history of endovascular aortic repair (EVAR) with Gore Excluder Polytetrafluoroethylene (PTFE) for a 6-cm infrarenal abdominal aortic aneurysm (AAA) presented 3 years later with insidious, deep abdominal pain. He had a history of diabetes, hypertension, and polymyalgia rheumatica. The physical exam was negative, including the abdominal exam. Labs and computed tomography (CT) imaging revealed nonspecific periaortic changes and small fluid collections, but no other pathology or signs of systemic infection were detected (Fig 1). Multiple cultures and white blood cell (WBC) scans were negative. CT-guided biopsy yielded serosanguinous fluid; culture, pathology, and stains for organisms were unrevealing. All aspiration and biopsy samples were obtained from the most representative and clearly pathologic areas within the peri-aortic retroperitoneum.

However, serology revealed elevated Phase I IgG titers (>1:1024) for *C. burnetii*, suggesting chronic Q fever. Molecular polymerase chain reaction (PCR) on blood and aspirates was inconclusive. The patient was started on doxycycline and hydroxychloroquine, with symptom resolution. Five months later, symptoms recurred, and CT imaging showed progression (Fig 1). Two-stage surgery was performed—axillo-bifemoral bypass, followed by graft explant and debridement. During the surgery in the retroperitoneum, dense inflammatory changes were encountered, without purulence. Serologic titers declined over time, indicating disease resolution. He completed 18 months of antibiotic therapy.

### Case 2 – *Bartonella henselae* infection

**Fig 2:**
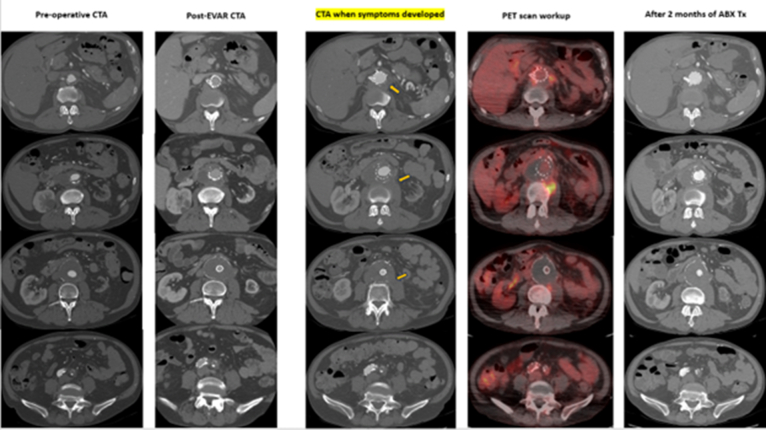
Captured timeline of the delayed development of *Bartonella henselae* endograft infection after endovascular aortic repair (EVAR). Arrows show enlarged retroperitoneal lymph nodes, which were confluencing, covering the abdominal aortic aneurysm (AAA) sac and neck near circumferentially. Positron emission tomography (PET) scan was positive. Antibiotic therapy (*ABX Tx*) was ineffective. *CTA*, Computed tomography angiography; *WBC*, white blood cell.

A 66-year-old man with a small AAA, renal insufficiency, and history of smoking presented with right groin and testicular pain. Imaging revealed AAA expansion to 5.6 cm and retroperitoneal lymphadenopathy. He underwent successful EVAR with Gore Excluder PTFE with renal artery stenting (Fig 2).

Six months later, he presented again with similar groin pain. CT showed circumferential peri-aortic lymphadenopathy. Positron emission tomography (PET)/CT revealed increased metabolic activity around the graft. Biopsy obtained from the peri-aortic retroperitoneum yielded serosanguinous material. Initial cultures were negative, but serology and PCR confirmed *B. henselae* (Fig 2).

Despite doxycycline and hydroxychloroquine, imaging showed no improvement. Surgical explantation with axillo-bifemoral bypass and retrograde renal artery bypass was performed. During the surgery in the retroperitoneum, inflammatory changes were encountered, but there was no purulence. PCR on the excised graft confirmed *B. henselae*.

## Discussion

Zoonotic vascular infections are rarely encountered by vascular surgeons and are challenging due to their indolent nature, lack of systemic signs, and culture-negative presentations. In both cases, conventional diagnostics failed; definitive diagnosis required a combination of serology, PCR, and PET/CT. To our knowledge, the *Bartonella*-associated endograft infection is the first reported case in the English literature. These fastidious organisms led to delayed diagnoses, as they were culture-negative and lacked classic infectious symptoms.

For *C. burnetii*, domestic ruminants (goats, sheep, cattle) are primarily the common reservoirs, and ticks are vectors, with transmission mainly via inhalation of aerosolized material (eg, contaminated dust, placental tissues). The common presentation is acute Q fever (flu-like illness in 50%), of whom 5% progress to chronic Q fever, which may present as culture-negative endocarditis or vascular infections months after exposure. In terms of *B. henselae* (cat scratch disease), domestic cats, particularly kittens, are primary reservoirs, and fleas (and possibly ticks or mosquitoes), are vectors, with transmission also via scratches or bites. Most cases are subacute or asymptomatic; severe disease may include lymphadenopathy, splenomegaly, hepatic lesions, endocarditis, and rarely vascular infections.

The first patient developed symptoms 3 years post-EVAR, and the second patient 6 months post-EVAR. Both patients were asymptomatic in the interim. Neither patient had systemic signs such as fever, malaise, weight loss, anorexia, diarrhea, gastrointestinal bleeding, or abnormal abdominal exams. The sole unifying symptom was localized, unexplained pain—deep abdominal pain in the first case, and flank/groin pain in the second.

*C. burnetii* has been implicated in vascular graft infections, but *B. henselae*-associated endograft infection has not previously been reported.^15^, ^16^, ^17^ Both pathogens display intracellular tropism and can persist despite immune responses and empirical antibiotics. Negative cultures and WBC scans may mislead clinicians. High suspicion, especially in patients with animal exposure or unexplained peri-aortic inflammation, is vital. In our two cases, the first patient resided near a dairy farm, a known risk factor for *Coxiella* exposure. The second patient was homeless, and had significant cat exposure, both of which are associated with *Bartonella* infection. Targeted diagnostics and early infectious disease consultation can expedite diagnosis.^18^, ^19^, ^20^ In both cases, medical therapy alone failed. Surgical explantation with extra-anatomic bypass was necessary to achieve source control and long-term resolution.^21^^,^^22^ Both patients underwent extensive retroperitoneal debridement of the inflamed, thickened tissue surrounding the aneurysmal aorta. The native aortic wall, which was loose and dissected, was also removed. Because of concerns about placing graft material and creating suture lines within an infected field, we opted for axillobifemoral bypasses followed by graft explanation in both cases. Both patients received long-term doxycycline and hydroxychloroquine, as recommended by the infectious diseases (ID) team. In the first patient, antibiotic therapy was continued for 18 months. Q fever serologies improved over time (Phase I IgG: 2048 at 21 months, 1024 at 27 months, and 512 at 30 months), indicating disease resolution. The second patient developed chylous ascites, likely due to retroperitoneal lymphatic injury, which was managed with total parenteral nutrition, followed by a low-fat diet. He also developed chronic renal failure requiring dialysis due to his solitary functioning right kidney. He was discharged successfully but elected palliative care 6 months later and passed away.

Vaccination for *Coxiella* is available for high-risk populations.^23^ For patients with known acute Q fever and vascular prostheses, PET/CT screening and prophylactic antibiotics are recommended.^24^ Although no such guidelines exist for *Bartonella*, the similarities suggest similar management strategies could be beneficial. Beyond the standard vascular graft infection workup, we emphasize that clinical symptoms can be vague and nonspecific, and cultures are often negative. Rather than recommending a fixed panel, we propose two critical steps:

(1) Early ID consultation: These infections often require specialized testing and long-term follow-up. The diagnostic complexity exceeds the typical training of vascular surgeons, making early ID involvement essential.
(2) PET/CT imaging: In our cases, PET/CT was more sensitive than tagged WBC scans. This aligns with other studies recommending PET/CT as a screening tool for vascular prosthesis involvement, particularly in acute Q fever.

## Conclusions

Zoonotic vascular infections caused by *Coxiella* and *Bartonella* are rare, insidious, and often culture-negative. Awareness, early suspicion, and targeted diagnostics including serology, PCR, and PET/CT are essential. Definitive therapy may require surgical explantation in addition to prolonged antibiotic therapy. Vascular surgeons should remain vigilant for these atypical pathogens when managing patients with prosthetic vascular grafts and unexplained inflammation.

## Funding

None.

## Disclosures

None.
